# Rationale, design, and implementation protocol of an electronic health record integrated clinical prediction rule (iCPR) randomized trial in primary care

**DOI:** 10.1186/1748-5908-6-109

**Published:** 2011-09-19

**Authors:** Devin M Mann, Joseph L Kannry, Daniel Edonyabo, Alice C Li, Jacqueline Arciniega, James Stulman, Lucas Romero, Juan Wisnivesky, Rhodes Adler, Thomas G McGinn

**Affiliations:** 1 Department of Medicine, Section of Preventive Medicine and Epidemiology, Boston University School of Medicine, 761 Harrison Ave, Boston, MA 02119, USA; 2 Department of Medicine, Division of General Internal Medicine, Mount Sinai School of Medicine, 17 East 102nd St., New York, NY 10029, USA; 3 Department of Medicine, Hofstra North Shore-LIJ Medical School, 300 Community Dr, Manhasset, NY 11030, USA

## Abstract

**Background:**

Clinical prediction rules (CPRs) represent well-validated but underutilized evidence-based medicine tools at the point-of-care. To date, an inability to integrate these rules into an electronic health record (EHR) has been a major limitation and we are not aware of a study demonstrating the use of CPR's in an ambulatory EHR setting. The integrated clinical prediction rule (iCPR) trial integrates two CPR's in an EHR and assesses both the usability and the effect on evidence-based practice in the primary care setting.

**Methods:**

A multi-disciplinary design team was assembled to develop a prototype iCPR for validated streptococcal pharyngitis and bacterial pneumonia CPRs. The iCPR tool was built as an active Clinical Decision Support (CDS) tool that can be triggered by user action during typical workflow. Using the EHR CDS toolkit, the iCPR risk score calculator was linked to tailored ordered sets, documentation, and patient instructions. The team subsequently conducted two levels of 'real world' usability testing with eight providers per group. Usability data were used to refine and create a production tool. Participating primary care providers (n = 149) were randomized and intervention providers were trained in the use of the new iCPR tool. Rates of iCPR tool triggering in the intervention and control (simulated) groups are monitored and subsequent use of the various components of the iCPR tool among intervention encounters is also tracked. The primary outcome is the difference in antibiotic prescribing rates (strep and pneumonia iCPR's encounters) and chest x-rays (pneumonia iCPR only) between intervention and control providers.

**Discussion:**

Using iterative usability testing and development paired with provider training, the iCPR CDS tool leverages user-centered design principles to overcome pervasive underutilization of EBM and support evidence-based practice at the point-of-care. The ongoing trial will determine if this collaborative process will lead to higher rates of utilization and EBM guided use of antibiotics and chest x-ray's in primary care.

**Trial Registration:**

ClinicalTrials.gov Identifier NCT01386047

## Background

The benefits of evidence-based medicine (EBM) on the quality of clinical care and improved patient outcomes have not achieved their potential [1]. While numerous EBM guidelines based on high-quality research have been generated and disseminated, data on their uptake into daily clinical practice have often been disappointing due to the challenges of integrating EBM recommendations into the point-of-care [2]. As a result, EBM guidelines often end up as either cluttered paper on the wall of the medical office or idiosyncratic teaching points rarely altering clinical practice. Finding strategies to implement EBM at the point-of-care is critical as monitoring agencies and payers are increasingly using EBM guidelines as markers of quality care.

Clinical prediction rules (CPRs) are a type of EBM that uses validated rules for simple sign or symptom-based probability scores to risk stratify patients for specific prognoses and/or diagnostic assessments [3]. While many high-quality CPRs exist, they have not been regularly implemented for day-to-day care due to inaccessibility at the point-of-care, a problem even more pronounced in the age of electronic health records (EHRs). Our search of the literature found no evidence of attempts to integrate CPRs into EHRs in the ambulatory setting, and only one instance of proposed integration in the inpatient setting [4]. Two well-validated CPRs are the streptococcal pharyngitis (strep throat) and bacterial pneumonia CPRs [5-7]. The strep throat CPR uses five criteria (fever, swollen lymph nodes, tonsillar exudates, strep exposure, and recent cough) to estimate the probability of strep throat in a patient with a sore throat [5]. The pneumonia criteria uses five criteria (fever, tachycardia, crackles, decreased breath sounds, and absence of asthma) to estimate the likelihood of a bacterial pneumonia in the setting of a cough [7]. While both rules have been well-validated in the literature, their use at the point-of-care is suboptimal and new methods for incorporating them into the point-of-care are needed.

Clinical decision support (CDS) systems have been developed as platforms within EHRs to provide evidence at the point-of-care and change physician behavior [8]. In theory, CDS should seamlessly integrate EBM into EHR systems to support the physician in delivering efficient, effective care at the point-of-care, but surprisingly has had equivocal results in ambulatory care [9-13]. Prior attempts at integrating these EBM delivery platforms into EHRs may have been limited by the lack of usability testing of the CDS interface and inadequate provider training prior to use [14]. The lack of usability testing (*i.e.*, useable and usefulness testing) limits the ability to assess if CDS can be effectively integrated into clinical workflow (usable) or is something desired by the clinician (usefulness). This often forces clinicians to either alter their workflow or work around the CDS tool. The lack of provider training in assessing the usability and usefulness of CDS tools and therefore how to best incorporate these tools into workflow has also limited their penetration into clinical practice. As a platform for building EBM into EHRs, CDS could significantly improve clinical workflow and quality delivery by providing access to many well-validated frontline decision aids like CPRs that are currently underutilized.

We have developed an integrated clinical prediction rules (iCPR) clinical decision support program that incorporates two well-validated CPRs (Walsh CPR for Streptococcal Pharyngitis and the Heckerling CPR for Pneumonia) into an outpatient EHR system used by the providers of nearly 40% of the nation's patients. This article discusses the design, development, usability testing, training, and implementation of study.

## Methods/Design

The iCPR study was designed to test the feasibility and effectiveness of incorporating the strep throat and pneumonia CPRs into the EHR in a primary care practice. The two main aims supporting this goal were to assess adoption of the iCPR program in primary care and to assess the impact of the iCPR

### Prototype development

**Figure 1:**
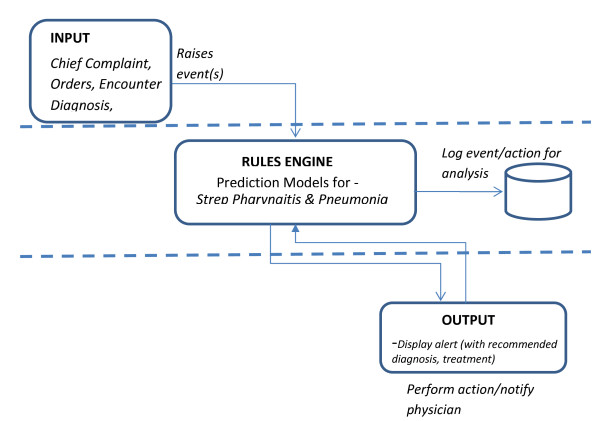
**iCPR conceptual model**.

Over a period of three months, an interdisciplinary team designed the first prototype iCPR. This team included expertise in CPRs, primary care, usability, clinical informatics, and a deep knowledge of the capabilities and limitations of CDS in the commercial EHR. Early in the prototype design process, several major design issues were considered. Figure 1 displays the basic conceptual model of the iCPR tool.

### Technical Considerations

#### Assessment Tool

We considered several options within the EHR to house the iCPR assessment tool based on discussions with the vendor, provider familiarity with the vendor's CDS tools, and provider workflow. The EHR vendor initially suggested using a 'smart' form for iCPR because it has enhanced visual aesthetics and expedites calculations. However, providers had almost no daily experience with this form in the practice under study and, more importantly, would have required more manual input by providers to complete the full iCPR workflow. As a result, the team selected to use dynamic flowsheets for calculations that were relatively unknown and had some formatting limitations, but minimized 'clicks' and manual data entry.

#### Restriction of alerts

iCPR is a practice-based randomized clinical trial that had to be seamlessly integrated into workflow without disrupting control providers. To achieve this, iCPR was designed to activate only for providers randomized to the intervention. Furthermore, the tool is further restricted to the providers' outpatient primary care EHR interface, because they may be practicing in other clinical settings with the same EHR but potentially vastly different workflows.

#### Alerts, overrides and triggers

Alerts are an active research area in the CDS literature. They can be categorized on two spectrums of activity: active versus passive and mandatory versus optional. A major early design consideration was the whether to use active (interrupting) or passive (non-interrupting) alerts [15,16]. In the context of our commercial EHR, active alerts 'pop-up' at the user, directly interrupting their workflow in order to draw their attention. Passive alerts are non-interrupting, minimally intrusive alerts, and would use non-interrupting flags or highlights to draw the CDS alert. Mandatory alerts require the user to take the designated action or explain the reason for overriding the CDS, while optional alerts allow the user to ignore the CDS alert without an explanation. Prior literature has demonstrated the superior efficacy of active mandatory alerts; however they are more disruptive to workflow, which contributes to the low uptake of CDS tools [10]. Their use is also problematic in the increasingly crowded CDS dashboards populating the primary care EHR. Balancing these factors, the development team selected a two-step system in which an early-in-workflow passive mandatory alert and a later-in-workflow active mandatory alert were combined. Mandatory alerts were chosen for both because data on reasons for declining the CDS tool were critical to iterative improvements.

Another major design issue was the choice of where in the primary care workflow the alert should launch and what the specific trigger diagnoses or orders should be. The pros and cons of various workflow triggers options were discussed and consensus was achieved for the initial prototype. The agreed trigger points for the tool were one of three workflow locations: chief complaint, relevant and specific encounter diagnoses, or a less specific encounter diagnosis in combination with a relevant antibiotic order (Table 1 lists the relevant trigger diagnoses and orders). The early-in-workflow passive mandatory alert triggered from the chief complaint, while the later-in-workflow active mandatory alert triggered from diagnosis and/or orders to ensure users did not simply forget to use the CDS tool.

**Table 1:** Chief complaint, diagnosis, and diagnosis/antibiotic combination triggers of iCPR tools

Strep	Pneumonia
**Chief Complaint**	
Sore throat	Possible pneumonia
Strep pharyngitis	Pleurisy
Dysphagia	Chest hurts when breathing
Throat hurts	Productive cough with shortness of breath
Throat discomfort	New onset shortness of breath
Recent contact (children) with pharyngitis	
**Diagnosis**	
Acute pharyngitis	Acute bronchitis
Bacterial pharyngitis	Acute bronchitis with bronchospasm
Chronic pharyngitis	Aspiration pneumonia
Difficulty in swallowing	Atypical pneumonia
Odynophagia	Bronchiectasis with acute exacerbation
Pain on swallowing	Bronchitis
Pharyngitis	Bronchitis with chronic airway obstruction
Pharyngitis acute	Bronchitis, chronic
Pharyngitis due to group A beta hemolytic Streptococci	Bronchitis, not specified as acute or chronic
Sore throat	CAP (community acquired pneumonia)
Sore throat (viral)	Community acquired pneumonia
Sore throat - chronic	Legionella infection
Sorethroat	LRTI (lower respiratory tract infection)
Strep sore throat	Pneumonia
Strep throat	Pneumonia, aspiration
Streptococcal pharyngitis	Pneumonia, community acquired
Streptococcal sore throat	Pneumonia, organism unspecified
Throat discomfort	Productive cough
Throat infection - pharyngitis	Sputum production
Throat pain	
Throat soreness	
Viral pharyngitis	
Diagnosis and antibiotic combination*	
Difficulty swallowing liquids	Abnormal breathing
Difficulty swallowing solids	Airway obstruction
Dysphagia	Allergic cough
Dysphagia, oropharyngeal	Breathing difficulty
Dysphagia, unspecified	Breathing problem
Esophageal dysphagia	Chest congestion
Impaired swallowing	Chest heaviness
Intermittent dysphagia	Chronic cough
Laryngeal pain	Chronic coughing
Pain on swallowing	Congestion pulmonary
Pain or burning when swallowing	Cough
Pain with swallowing	Cough due to angiotensin-converting enzyme inhibitor
Painful swallowing	Cough secondary to angiotensin converting enzyme inhibitor (ACE-I)
Problems with swallowing	Coughing
Swallowing difficulty	Cryptogenic organizing pneumonia
Swallowing disorder	DOE (dyspnea on exertion)
Swallowing impairment	Dry cough
Swallowing pain	Dyspnea
Swallowing pain or burning	Dyspnea on exertion
Swallowing painful	Exertional dyspnea
Trouble swallowing	Hypercarbia
	Non-productive cough
	Nonproductive cough
	Other dyspnea and respiratory abnormality
	Productive cough
	Pulmonary edema
	Recurrent upper respiratory infection (URI)
	Respiratory tract infection
	Shortness of breath
	Shortness of breath dyspnea
	Shortness of breath on exertion
	SOB (shortness of breath)
	Trouble breathing
	URI (upper respiratory infection)
	Viral bronchitis
	DOE (dyspnea on exertion)

*Antibiotics: Oral penicillins, macrolides, cephalosporins, quinolones, tetracyclines

#### Risk calculator

The development team next looked at which patient-specific elements of the history and physical exam (auto-generated when possible) the tool could use to automatically calculate the risk probabilities and provide recommendations suggested by validated CPRs. While several alternatives including traditional CDS templates were considered, it soon became clear that dynamic flowsheets would be used because this functionality would enable the required calculations of CPRs while maintaining the hub-and-spoke linkages critical to successfully integrating CDS tools into workflow [15].

#### Bundled order sets, documentation, and patient instructions

**Figure 2:**
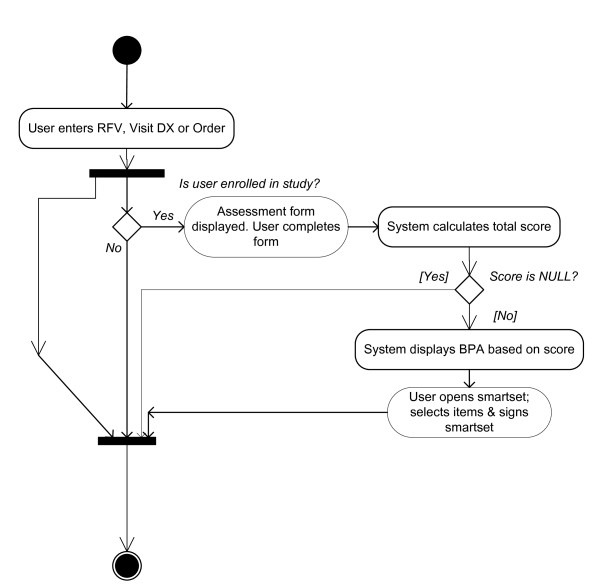
**Schematic flow of iCPR tool**.

The design specifications called for integrated bundled order sets, template documentation, and patient instructions that would be linked to each CPR in order to further enhance provider usability and buy-in. The team constructed bundled order sets tailored to each of the potential risk states calculated by the CPR tool. Three versions of the iCPR were created for strep throat--low-, intermediate-, and high-risk. Low risk led to a bundled order set without antibiotics, intermediate led to a workflow with rapid strep as the next step (with resulting low- or high-risk order sets), and high risk led to a bundled order set with pre-populated suggested antibiotic orders. The pneumonia iCPR had a similar format but with only low- and high-risk states. Clinical experts populated each bundled order set with the most common orders (antibiotics, symptom relief medications, *et al*.) used for strep throat and outpatient pneumonia treatment. They also guided the development of the clinical documentation that auto-populated the progress note of the visit, a key to enhancing the usability of the tool. Auto-generated patient instructions in English and Spanish were also developed for each risk state. The instructions outlined expected duration, etiology of the illness (viral or bacterial), triage steps for worsening symptoms, description of symptom relief medications, and contact information. Figure 2 represents a schematic flow of the iCPR tool. With the prototype iCPRs built, the team moved into the usability phase to evaluate the prototype's ability for workflow integration and for meeting the provider's preferences.

#### Usability testing

**Figure 3:**
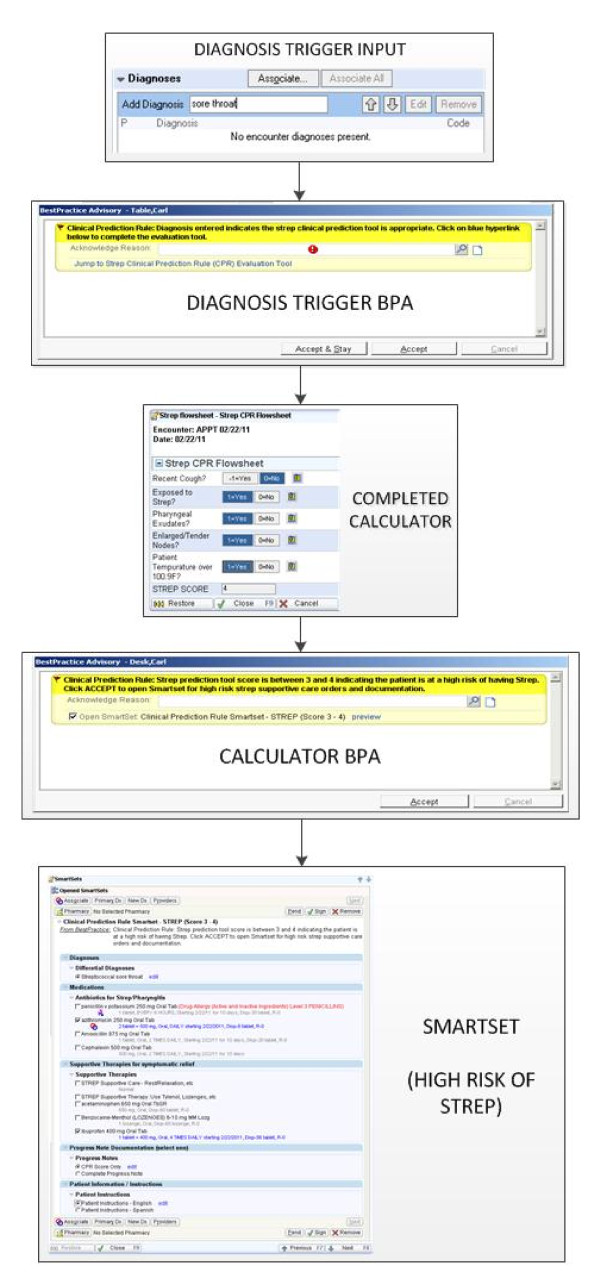
**Screenshots of finalized iCPR tool**.

**Figure 4:**
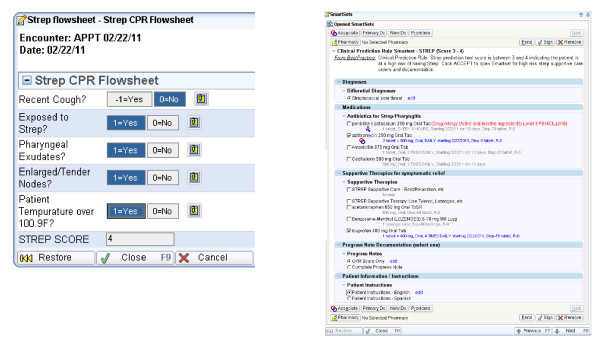
**Magnified views of risk score calculator and bundled order set**.

We conducted usability testing to evaluate the main functionalities of the iCPR tool: alerting, risk calculator, bundled ordering, progress note, and patient instructions. Using 'think aloud' and thematic protocol analysis procedures, simulated encounters with eight providers using written clinical scenarios were observed and analyzed. Screencapture software and audiotaping were used to record all human-computer interactions. Themes were reviewed by the study team, and consensus was used to guide prototype refinements when technically and logistically feasible. A second round of usability testing with eight additional providers was conducted using trained actors to simulate 'live' clinical encounters. These additional data were coded using a time-series analytic procedure that focused on the workflow of encounters to help understand issues not generated in the scripted 'think aloud' scenarios. A full description of the usability testing design and findings is described separately (in preparation). These data were then reviewed, and additional modifications were incorporated into the prototype to achieve the final iCPR tools. Figures 3 and 4 depict the finalized components of the iCPR tool.

### Trial design

#### Practice setting

The study was conducted at a large urban academic medical center. All of the providers were members of the academic primary care practice that is located on the main hospital campus. The outpatient clinic has over 55,000 visits annually and serves a diverse population that is approximately 56% Hispanic, 35% African-American, 7% white and 2% other.

#### Provider eligibility, consent, and randomization

All primary care providers within the medical practice were eligible for the study. The practice includes 149 primary care faculty, residents, and nurse practitioners divided into four units on the same floor. The study design was a randomized control trial in which the providers within the academic medical center outpatient practice were the unit of randomization. Faculty providers were randomized via random number generator to intervention or control in a 1:1 ratio. Medical residents were randomized within blocks according to their outpatient ambulatory care month (a period with substantially increased outpatient clinical activity) assignments to ensure even distribution throughout the academic calendar. However, due to changes in the resident calendar in year two of the study, any additional medical resident providers entering the system were added in a 1:1 fashion. Only providers randomized to the intervention are triggered by the EHR to use the iCPR tools. After randomization, all providers were invited to standardized educational forums for consent and training (if randomized to the intervention).

#### Provider Training

All providers allocated to the intervention received approximately 45 minutes of training on how the iCPRs are integrated into the EHR and how to interpret the output of each iCPR. Each training session was led by at least one study investigator and one study staff member. The training consisted of a background on the strep throat and pneumonia CPR evidence, several walkthroughs of iCPR tools using the EHR training version, and a demonstration video simulating the tool in a live clinical encounter. Providers who were unable to attend group training sessions were trained individually.

#### Patient inclusion and exclusion criteria

There was no specific patient inclusion/exclusion criteria used in iCPR. The initial plan had been to use age, prior hospitalization history, and current/recent antibiotic use as criteria, but these were eliminated due to a variety of reasons, including inadequate/inaccurate documentation of prior medical history and current medication prescription. Thus, other than being an enrolled intervention provider, the only criteria for inclusion were the appropriate triggering diagnoses, chief complaint, or a diagnosis/order combination. The list of chief complaints and related diagnoses and orders that trigger each iCPR is listed in Table 1. Common triggers include a chief complaint or diagnosis of 'sore throat' for the strep throat iCPR and a diagnosis of 'bronchitis' for the pneumonia iCPR.

### Measures

#### Baseline

##### Patient level

Patient characteristics, including age, gender, comorbidities, smoking history, recent hospitalizations and current or recent medications, are captured via EHR chart review.

##### Provider level

Provider characteristics including age, gender, and years of practice are captured via self-report.

### Follow-up

#### Process

The process measurement battery is designed to assess the uptake of the iCPR tool by providers and to document the utilization of each part of the tool. This is a critical outcome because poor provider utilization of CDS and other EBM and quality improvement tools has been a frequent barrier to their success [9]. Measured markers of utilization (see Table 2) include rate of accepting the iCPR tool when triggered in an encounter, using the relevant iCPR risk calculator, use of the bundled order set linked to each risk calculator score, and use of each section of the bundled order set (orders, documentation, patient instructions, *et al*.). The rate of triggering of the iCPR tool from the various sections of the EHR will be measured in the intervention and control arm. The control arm is measured through 'shadow' simulation of the iCPR tool in the control patients, which allows comparison of triggering rates in the control and intervention.

**Table 2:** Outcome measures

CPR	Process Outcomes	Primary Outcome	Secondary Outcomes
Pneumonia	% of eligible encounters accepting iCPR and using bundled order set	Number of antibiotics prescribed	Number of chest x-ray ordered
Strep throat	% of eligible encounters accepting iCPR and using bundled order set	Number of antibiotics prescribed	Number of rapid strep tests and throat cultures ordered

#### Outcome

The outcome measurement battery is designed to detect changes in clinical practice that are most likely to result from use of the iCPRs. The primary outcome is the difference in antibiotic prescribing frequency among patient encounters eligible for the iCPR tool among intervention compared to control providers. For example, for all patients presenting with symptoms that launch the pneumonia or strep throat tools, data will be collected from the EHR on the number of prescriptions for antibiotics written by providers randomized to the iCPR compared to usual-care arms, respectively. We will also examine the rate of chest x-ray orders and rapid strep throat test orders between intervention and control providers as a secondary outcome (see Table 2).

#### Data monitoring and quality control

All data collection is conducted via the EHR. Weekly reports are generated to track the frequency of the tool triggering, including the use of each component of the iCPR tools and the respective diagnostic triggers. Periodic chart reviews are conducted to monitor the appropriateness of tool triggering and to investigate any concerns raised by providers regarding usability or workflow disruptions. In addition, provider refresher training is conducted prior to residents coming onto each subsequent ambulatory care block in order to maintain a consistent ability to use the tool. The refresher consists of a videoclip simulation of a provider and patient interacting with tool.

### Statistical Analysis

The planned statistical analyses include comparing socio-demographic and other baseline characteristics. Patient comparisons will be conducted by stratifying the sample by randomization status and by condition (*i.e.*, pharyngitis and pneumonia). We will use the t-test, Wilcoxon test, or the chi-square test, as appropriate, to evaluate the balance between groups. The relative frequency of triggering of each iCPR in intervention and control patients, overall, and by where the triggering occurs (chief complaint, ordering, or diagnosis) will then be compared. We will calculate the proportion of intervention encounters in which each component of the iCPR tool, including the overall tool, the risk calculator, and the bundled order set, are used. This calculation will be repeated stratifying by test condition and by provider characteristics (training level, *et al*.). To test the effect of iCPR, we will use a generalized estimating equation model with clinician as the cluster variable, antibiotic prescribing as the outcome variable, and intervention group as the only explanatory variable. Given the nature of the possible relationship between patients in a cluster, we will use an exchangeable correlation structure for parameter estimation.

### Power Calculation

Sample size was calculated as if individuals were independent, and then adjusted to account for the clustering of patients within physicians. Although patient outcomes are assumed to correlate somewhat within provider, the multicausal nature of clinical outcomes and the likely random nature of patient assignment to providers led us to estimate a small interclass correlation (intraclasss correlation coefficient for binomial response < 0.15). The calculation of sample size was performed with a significance level of 0.05 and 80% power. The adjusted sample size was calculated by multiplying the initial estimate of the number of patients by an inflation factor, which is a function of the interclass correlation and the number of the clusters. Final calculations estimated a need of 1,070 study subjects (535 in each disease condition) in total assuming a baseline rate of 30% antibiotic ordering in each condition and an estimated effect size of a 12% reduction in ordering in the intervention arm.

### Implementation

Several steps were taken to ensure a smooth and successful implementation of the iCPR CDS. A rapid response team composed of informatics and clinical expertise was available via pager for the first week after roll-out to respond to early bugs and other issues in real time. In addition, the team later embedded an option into iCPR for users to send messages to the build team to communicate issues. Furthermore, the lead clinician maintained a 'presence' in the practice so that any building frustration or problems with the tool could be handled rapidly before it built into more substantial resistance. Lastly, periodic focus groups were held to elicit users' feedback on the tool; these data were used to conduct ongoing refinements. The study was launched in December 2011 and is ongoing.

## Discussion

The iCPR trial was designed to assess whether a highly integrated CDS tool that supports clinicians in making EBM guided decisions is feasible, accepted, and effective. The team composition and design choices throughout the development process reflect the project's focus on enhancing provider acceptance and usability. The tool was designed by a multi-disciplinary development team that encouraged clinician users and designers to work together from inception. Iterative, *in vivo *usability was another key towards enhancing clinician acceptance because the think aloud and trained actor 'live' simulations each provided feedback that substantially improved the prototype. This approach differs from the more traditional usability testing under carefully controlled conditions that often minimizes the input of actual users in a realistic use setting [17]. Standardized training demonstrated the new workflows to all intervention clinicians; another likely contributor to broad acceptance of the tool. Too often, new tools are rolled out into production with suboptimal training, creating resistance among providers [18]. In summary, we believe that this 'grassroots' approach paired with usability and user training will improve previously disappointing update of similar CDS tools [9]. The overall acceptance of the tool and its ability to alter antibiotic prescribing for suspected strep or pneumonia will be determined by the final outcomes of the trial. However, the approach used serves as a model for a more user-centered design of CDS; one that maximizes provider input and likely acceptance. These lessons should be generalized more broadly in CDS development of EBM and other point-of-care CDS tools.

## Competing interests

The authors declare that they have no competing interests.

## Authors' contributions

DMM conceived the study concept, protocol and design, supervised implementation and coordination, conducted analyses, and drafted the manuscript. JLK conceived the study concept, protocol and design, supervised implementation and coordination, conducted analyses, and drafted the manuscript. DE helped design the prototype and study protocol, trained providers, conducted analyses, and revised the manuscript. ACL helped develop the study protocol and prototype, trained providers, and supervised implementation. JA helped conceive the study protocol and design, supervised implementation, and provided study coordination. LR helped supervise implementation and data collection, trained providers, and revised the manuscript. JS supervised implementation and coordination, trained providers, and reviewed analyses. JW helped conceive the study design, supervised coordination and implementation, and supervised analyses. RA helped with study implementation and trained providers. TPM conceived the study concept, protocol and design, supervised implementation and coordination, and help draft the manuscript. All authors read and approved the final manuscript.
